# Dynamics of RIF1 SUMOylation is regulated by PIAS4 in the maintenance of Genomic Stability

**DOI:** 10.1038/s41598-017-16934-w

**Published:** 2017-12-12

**Authors:** Ramesh Kumar, Chit Fang Cheok

**Affiliations:** 1 IFOM-p53Lab Joint Research Laboratory, 8A Biomedical Grove, #06-38, Immunos, A*STAR, S138648 Singapore, Singapore; 2 IFOM, The FIRC Institute of Molecular Oncology Foundation, Via Adamello 16, 20139 Milan, Italy; 3 Department of Biochemistry, Yong Loo Lin School of Medicine, National University of Singapore, Singapore, S117597 Singapore; 4 Department of Pathology, Yong Loo Lin School of Medicine, National University of Singapore, Singapore, S119077 Singapore; 5 Cancer and Stem Cell Biology Program, Duke–NUS Graduate Medical School, 8 College Road, Singapore, 169857 Singapore

## Abstract

RIF1 plays a key role in inhibiting DNA end resection and promoting NHEJ mediated DNA double stand break repair in G1. However, whether SUMOlyation may regulate RIF1 functions is still largely unknown. Here, we report that RIF1 is SUMOlyated in response to DNA damage. We identified PIAS4 as the primary SUMO E3 ligase required for the SUMOylation of RIF1 protein. Mammalian cells compromised of PIAS4 expression, show impaired RIF1 SUMOylation and defective for the disassembly of DNA damage responsive RIF1 foci. Mechanistically, we show that PIAS4 knockdown abrogates UHRF1-dependent ubiquitination of RIF1, compromising RIF1 protein turnover. We detected intense RPA foci that colocalize with RIF1 foci in PIAS4 knockdown cells. These data highlight an important role of PIAS4-dependent regulation of RIF1, likely mediated by SUMOylation, in the disassembly of RIF1 DNA damage response (DDR) foci. We propose that unresolved RIF1 protein at sites of DNA damage in PIAS4-depleted cells largely accumulates in S phase, and subsequently leads to DNA double strand breaks. Therefore, PIAS4 promotes genomic stability by regulating the timely removal of RIF1 from sites of DNA damage.

## Introduction

DNA damage activates a wide range of responses including altered gene expression, cell cycle arrest and activation of DNA repair^1^. To preserve genome integrity after genotoxic insult, eukaryotic cells have developed a highly conserved surveillance mechanism, collectively termed the DNA damage response (DDR) pathway^2,3^. In response to DNA double strand breaks (DSBs), components of DDR signaling drive two main repair pathways, NHEJ and HR^4,5^. In G1 cells, in the absence of sister chromatid and inadequate CDK activity, nucleolytic resection of 5′ end is inhibited, which in turn promotes the 53BP1-mediated NHEJ break processing^6^. However, in S and G2 phases, CDK phosphorylation of BRCA1/CtIP drives the 5′–3′ DNA end resection which facilitates the HR process to repair the DNA DSBs^7^.

PTMs involve (but not limited to) phosphorylation, methylation, acetylation, SUMOylation and Ubiquitination. In the latter two PTMs, Ubiquitin and SUMO polypeptides are covalently attached to target protein via isopeptide linkage^8,9^. The extent of SUMO modifications of the target proteins depends on the number of SUMO conjugation. Some of the target proteins have a single SUMO attached, while in others, multiple Lys residues on the target are individually linked to SUMO^10,11^.

Coordinated PIAS1 and PIAS4 mediated protein SUMOylation and ubiquitination facilitate the distribution of DDR components (MDC1, BRCA1 and 53BP1) at the sites of DNA breaks and promote the repair process^12^. SUMOylation deficient mouse embryos die early due to defective chromosomal segregation, suggesting a key role for SUMO in maintaining genomic integrity^13,14^. It has been established that SUMO conjugates, SUMO-conjugating enzymes UBC9 (UBE2I) and SUMO E3 ligases, PIAS1 (protein inhibitor of activated STAT 1) and PIAS4 (PIASy), are recruited at sites of DSB, which in turn promote DSB signaling and repair^12,15^. PIAS4 mediates SUMO-2 conjugation of Topoisomerase-II on mitotic chromosomes^16^. SUMO2 modification of Rev1 by PIAS4 regulates p53-dependent cancer cell death in response to oxidative stress^17^. Elegant works from different laboratories indicates that PIAS1 and PIAS4 function in parallel but overlapping SUMO-conjugation pathways to facilitate the DNA break repair^12,15^. Previous studies have also detected SUMOylated 53BP1 in His purified SUMO2 conjugates and unlike BRCA1 and MDC1, SUMOylated 53BP1 was not increased after RNF4 knockdown^18^. Earlier studies have revealed a function for SUMO and ubiquitin in the recruitment and disassembly of DNA repair foci to prevent genomic instability^19–22^.

Identification of RIF1 at the sites of DNA breaks was reported previously^23–25^. However, its broader function in the regulation of key DNA repair process has only recently been evidenced. RIF1 has been identified as an effector of 53BP1, which modulates the DNA DSBs repair by regulating NHEJ in G1 cells. In contrast, during S/G2 phase of cell cycle, BRCA1-CtIP mediated DNA end resection prevents NHEJ through the removal of 53BP1-RIF1 from DSBs^26–31^. Several earlier reports have demonstrated novel functions of RIF1 in the maintenance of genomic stability, replication timing, nuclear architecture, class switch recombination and immunological functions^32–36^. RIF1 is a large nuclear protein. It’s molecular and biochemical basis of action and its upstream regulation is still unclear. BLM and RIF1 interact physically and are recruited at the stalled replication fork with similar kinetics^37^. In addition, BLM SUMOylation is required for RAD51 localization at damaged replication forks and repair by HR^38,39^.

In this study we report that RIF1 is regulated by SUMOylation in response to DNA damage. We identified PIAS4 as the main SUMO E3 ligase required for RIF1 SUMOylation. PIAS4 deficient mammalian cells showed impaired RIF1 SUMOylation and defective disassembly of RIF1 DDR foci after recovery from DNA damage. These RIF1 foci resulted in increased replication stress and DNA double strand breaks. Moreover, we noticed multiple RIF1 and 53BP1 nuclear bodies in PIAS4 depleted cells. Overall, we have identified RIF1 as a novel PIAS4 target protein required for the maintenance of genomic integrity.

## Results

### RIF1 SUMOylation is increased in response to DNA double strand breaks

**Figure 1:**
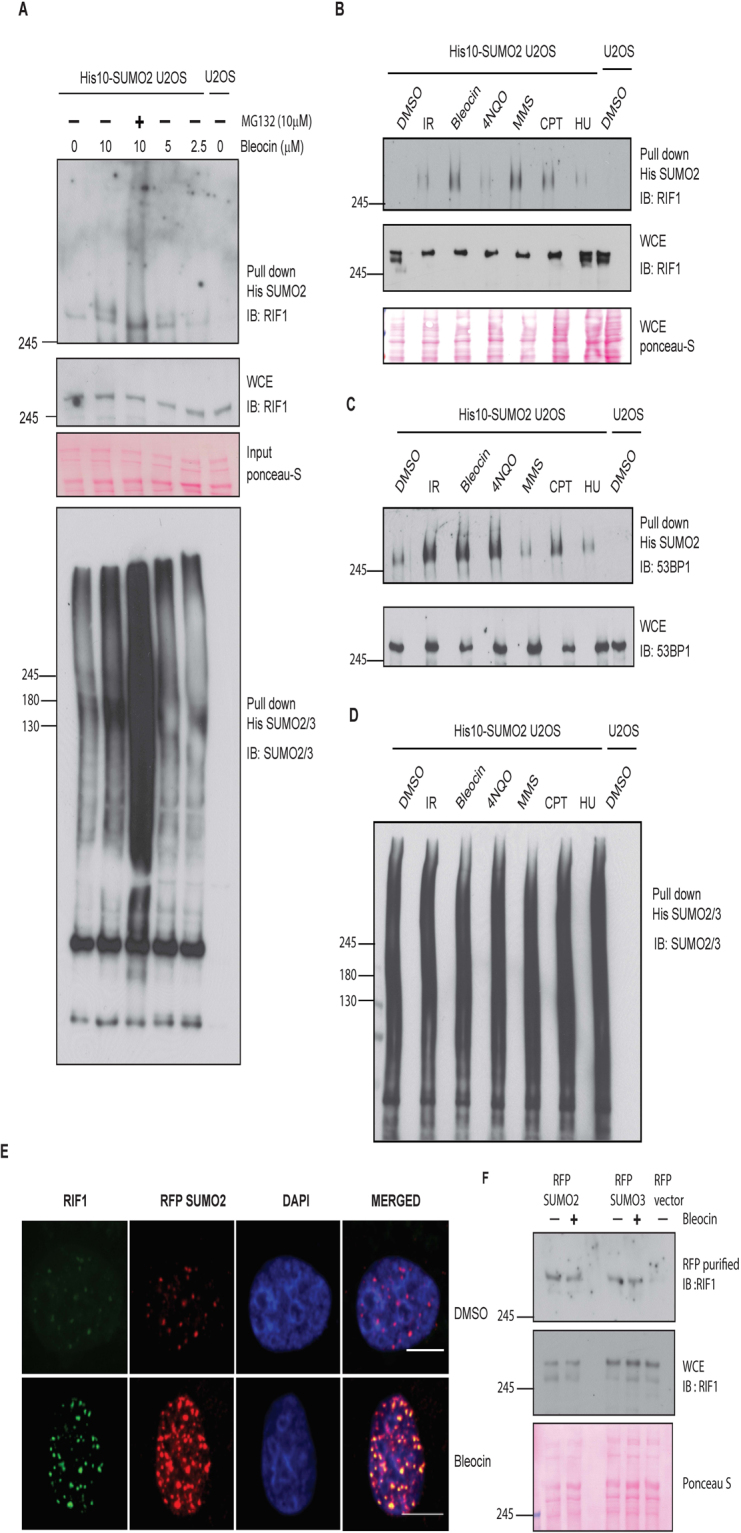
RIF1 is SUMOylated protein. (**A**) U2OS cells stably expressing 10 His-SUMO2 were either DMOS treated or treated with indicated doses of bleocin and MG132. Cells were harvested and SUMO2 protein conjugates were purified from denaturing lysates. Whole cell lysate (WCE) and His purified SUMO2 conjugates were immunoblotted with antibodies directed against RIF1 and SUMO2/3. Additionally, parental U2OS cells were included in all experiments, as a negative control. (**B**) U2OS cells stably expressing 10 His-SUMO2 were either DMOS treated or treated with the indicated DNA damaging agents. His purified SUMO2 conjugates and WCE were immunoblotted with RIF1 antibody. (**C**) Protein samples from Fig. 1B were immunoblotted with antibody directed against 53BP1. (**D**) Total SUMO2/3 in His purified SUMO2 conjugates were detected using SUMO2/3-specific antibody. (**E**) Colocalization between RIF1 and SUMO2 foci. U2OS cell were transiently transfected with RFP-SUMO2 construct and treated with bleocin. Cells were immunostained with RIF1 antibody and subsequently stained with Alexa Fluor 488 secondary antibody. Horizontal bar represents 10 μm. (**F**) U2OS cells transfected with RFP-SUMO2, RFP-SUMO3 and RFP empty vector constructs. Cell were mock treated or treated with low dose (2.5 µg/ml) of bleocin. RFP-tagged proteins and associated protein conjugates were purified using RFP-Trap® assay and immunoblotted with RIF1 antibody.

The increasing importance of SUMOylation in the regulation of DDR response and protein dynamics at DNA breaks prompted us to investigate the role of RIF1 SUMOylation in the regulation of RIF1 functions. To detect RIF1 SUMOylation *in vivo*, we have used a U2OS cell line stably expressing 10 His SUMO2^40,41^. DMSO or bleocin treated cells were lysed and His purified SUMO2 protein samples were immunoblotted to detect SUMOylated RIF1 protein. We noted a distinct slower migrating form representing SUMOylated RIF1. Interestingly, SUMOylated RIF1 protein signal was further increased in response to bleocin and MG132 treatment (Figs 1A and S1A). We further tested the specificity of DNA damaging agents (listed in Table 1), triggering RIF1 SUMOylation and we noticed an enhanced SUMOylated signal in response to all genotoxic agents included in this study (Fig. 1B). We noticed a better RIF1 SUMOylation signal in response to bleocin and MMS treatment. Similar to RIF1, SUMOylation of 53BP1 was also increased in response to different forms of DNA damaging agents (Figs 1C and S1B). His purified SUMO2 conjugates were blotted with SUMO2/3 antibody to determine the total level of SUMO2 in different protein samples (Figs 1A, S1A, 1D and S1B lower panels).

**Table 1:** List of drugs.

Drugs	Supplier	Cat. No.
Bleocin	Millipore	203401-10MG
Captothecin	Singma	C9911-1G
4NQO	Sigma	N8141
HU	Sigma	H8627
MMS	Sigma	129925
MG132	Millipore	474790
DMSO	Sigma	472301
Thymidine	Sigma	T9250

RIF1 is recruited at sites of DNA damage and colocalize with phosphorylated H2AX (pS139)^24,42^. Earlier reports have also shown that 53BP1 and BRCA1 colocalize differentially with SUMO isoforms at the local site of DNA damage^12,15^. We found that the majority of RIF1 foci colocalize with SUMO2 (Fig. 1E). In contrast, only a small fraction of RIF1 foci colocalize with SUMO1 (Fig. S1C). To confirm our cell biology data, RFP fused SUMO2 and SUMO3 were analyzed for their ability to bind RIF1. Interestingly, we detected RIF1 in RFP-SUMO2 and SUMO3 purified protein complexes (Fig. 1F). Taken together our results indicate that RIF1 is a SUMO modified protein and SUMOylation of RIF1 is increased in response to a broad range of DNA damaging agents.

### RIF1 SUMOylation is increased in G1 cells

**Figure 2:**
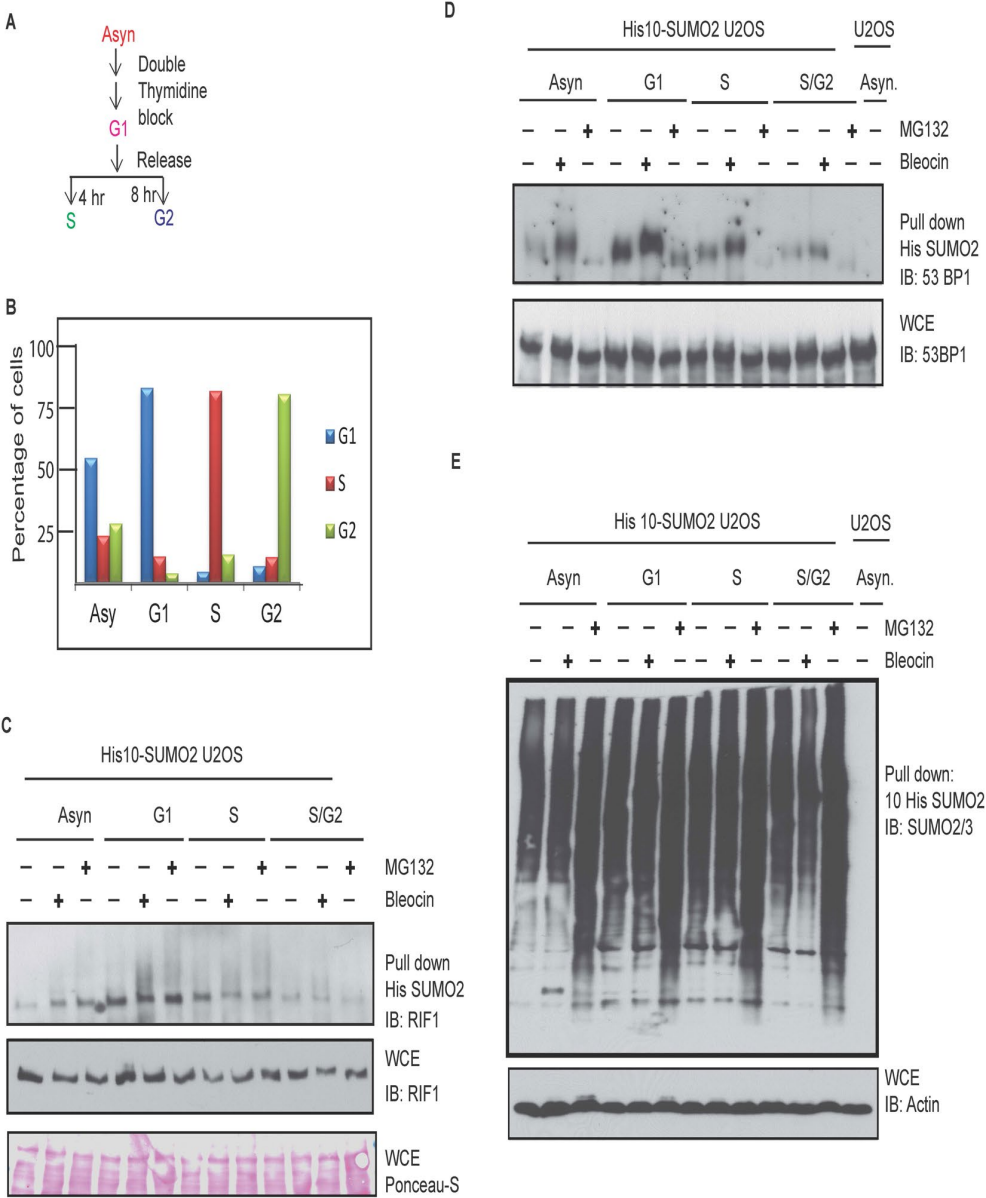
Cell cycle dependent RIF1 SUMOylation is increased in G1 cells. (**A**) Schematic diagram of the cell cycle synchronization and release experiment. (**B**) Graph represents the percentages of G1, S and G2/M cell populations determined by flow cytometry. (**C**) Timed release of G1 synchronized His10-SUMO2 expressing U2OS cells from double thymidine block followed by treatment with bleocin and/or MG132. His purified SUMO2 conjugates and WCE were immunoblotted with RIF1 antibody. (**D**) His purified protein samples from the Fig. 3C  were immunoblotted with 53BP1 antibody. (**E**) His purified protein samples and WCE were immunoblotted with SUMO2/3 and actin antibody respectively.

To determine the cell cycle specificity of the RIF1 SUMOylation, cell synchronization experiments were performed (Fig. 2A). Flow cytometry data confirms the enrichment of cells at different cell cycle phases (Fig. 2B). Interestingly, a clear enhancement of SUMOylated RIF1 signal was observed in G1 cells, which was further increased in response to bleocin treatment (Fig. 2C). RIF1 SUMOylation was decreased in S and G2 cells, confirming the specificity of RIF1 SUMOylation in G1. Similar to RIF1 SUMOylation, DNA damage induced 53BP1 SUMOylation was also enriched in G1 cells (Fig. 2D). His purified SUMO2 conjugates were immunoblotted with SUMO2/3 antibody (Fig. 2E), which demonstrated that the levels of purified protein complexes are similar in different samples.

### SUMO E3 ligase PIAS4 regulates RIF1 SUMOylation

**Figure 3:**
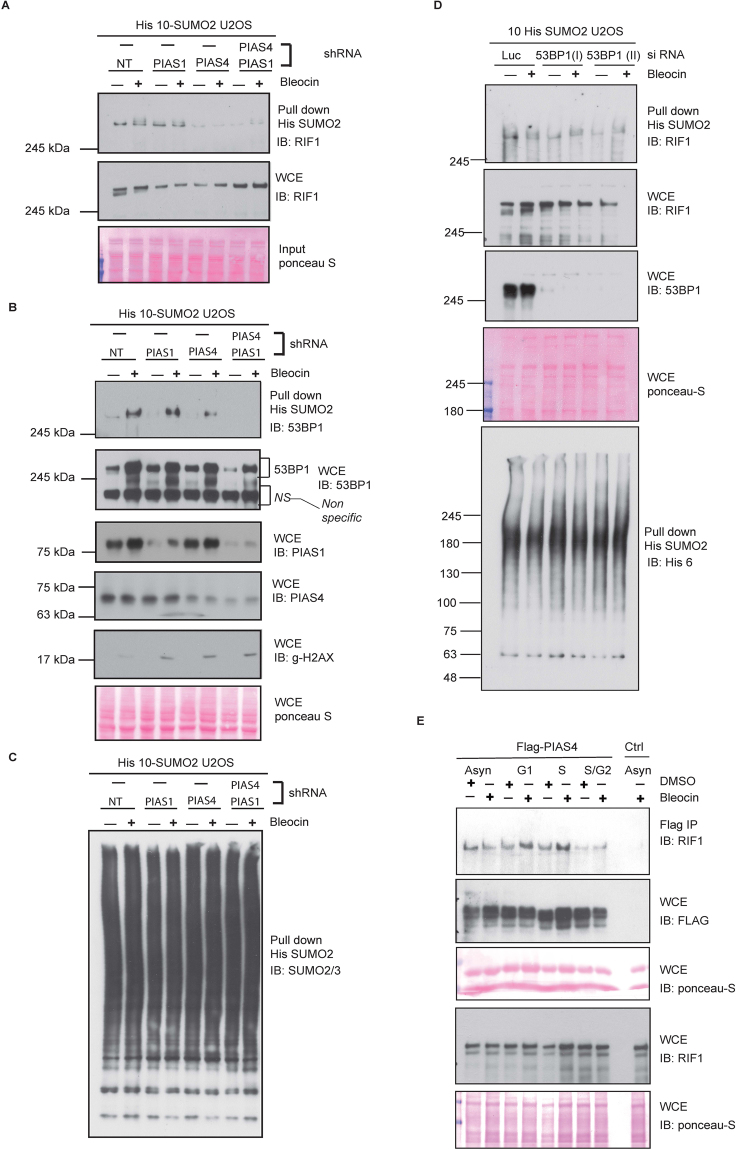
SUMO E3 ligase PIAS4 regulates RIF1 SUMOylation. (**A**) U2OS cells stably expressing 10 His-SUMO2 were either transfected with control shRNA or PIAS1 and PIAS4-specific shRNAs, and were treated with bleocin or DMSO. His-SUMO2 conjugates were purified and immunoblotted with RIF1 antibody. Protein samples from WCE were immunoblotted with RIF1 antibody. (**B**) Protein samples from the above experiment were analyzed for 53BP1 SUMOylation. WCE were probed with 53BP1, PIAS1, PIAS4 and y-H2AX antibodies. (**C**) His purified SUMO2 conjugates from (A) were immunoblotted with SUMO2/3 antibody to detect total SUMO2/3. (**D**) U2OS cells stably expressing His10-SUMO2 were either transfected with control siRNA or with two independent siRNAs targeting 53BP1. 3 days later, cells were treated with bleocin or DMSO as control. His purified SUMO2 conjugates and WCE were immunoblotted with RIF1 antibody. WCE was immunoblotted with 53BP1 antibody. His purified SUMO2 conjugates were immunoblotted with anti-6XHis tag antibody to establish equivalent His pull-down efficiency in all conditions. (**E**) U2OS cells were transiently transfected with FLAG-PIAS4 and synchronized at G1, S and G2. Flag-IP samples were probed with RIF1 antibody to determine the RIF1-PIAS4 bindings in different cell cycle stages. Whole cell lysates were immunoblotted with RIF1 and Flag antibody to determine the total proteins.

Increasing evidences suggest that members of the protein inhibitor of activated signal transducer and activator of transcription (PIAS) family of SUMO E3 ligases play crucial roles in the maintenance of genomic integrity. To identify the SUMO E3 ligase required for RIF1 SUMOylation, we first depleted cells of PIAS1 and PIAS4 by using specific RNAi sequences (listed in Table 2). Interestingly, we noted a substantial reduction of RIF1 SUMOylation specifically upon PIAS4 depletion (Fig. 3A), whereas PIAS1 depletion affected only partially the extent of RIF1 SUMOylation (Figs 3A and S3A). This indicates a predominant role of PIAS4 in regulating RIF1 SUMOylation. Consistent with an earlier report^18^, our data also suggest that SUMO2 modification of 53BP1 is significantly increased in response to DNA double strand breaks (Figs 1C, 2B and S1B). Here, we further demonstrated that PIAS4 is also required for SUMO-2 modification of 53BP1. PIAS4 depletion significantly reduced the level of SUMOylated 53BP1 and residual SUMOylation signal was abolished after PIAS1 and PIAS4 co-depletion (Fig. 3B). Total SUMO2 proteins in purified complexes were determined by SUMO2/3 (Figs 3C and S3A) or 6xHis (Fig. S3B) immunoblotting.

**Table 2:** List of Si RNA sequences.

S. No.	siRNA	source	sequence
1	PIAS1/I	SI00113974 QIAGEN	GGAUCAUUCUAGAGCUUUA
2	PIAS1/II	dharmacon	CGAAUGAACUUGGCAGAAA
3	PIAS4/I	SI00684439 QIAGEN	GGAGUAAGAGUGGACUGAA
4	PIAS4/II	Dharmacon	AGGCACUGGUCAAGGAGAA
5	Luc	Dharmacon	CGUACGCGGAAUACUUCGA
6	UHRF1 smartpool	Dharmacon	E-006977-00-0005 5
6	53BP1	Dharmacon	GAAGGACGGAGUACUAAUA
7	53BP1 smartpool	Thermo scientific	L-003548-00

Given that phosphorylation of 53BP1 was reported to be important in the regulation of RIF1 functions in NHEJ^27^, we asked if 53BP1 is directly required for RIF1 SUMOylation. However, we did not observe any substantial reduction of SUMOylated RIF1 signal in 53BP1-depleted cells (Fig. 3D), suggesting that RIF1 SUMOylation is not regulated by 53BP1. Whole cell lysates and His purified SUMO2 conjugates were immunoblotted for His6 (Fig. 3D lower panel) to determine the total level of proteins.

To understand the molecular basis of PIAS4 dependent RIF1 SUMOylation, we analyzed the extent of RIF1-PIAS4 interaction in cells and asked whether that may be cell cycle-regulated. U2OS cells were transiently transfected with FLAG-PIAS4 plasmid DNA and cells were enriched in different cell cycle phases, using the same synchronization protocol as depicted in Fig. 2A. Clearly, we observed an increased interaction between RIF1 and PIAS4 in G1-enriched cells (Fig. 3E) as well as in S phase enriched cells. In contrast, a reduced RIF1-PIAS4 binding was detected in G2-enriched synchronized cells. This result provided evidence that support our earlier findings that RIF1 is mainly SUMOylated in G1 cells (Fig. 2C). Additionally, the increased PIAS4-RIF1 interaction in S phase cells, which was further enhanced in response to DNA damage, suggest other potential roles of PIAS4 in regulating RIF1 in replicating cells.

### PIAS4 is required for the resolution of RIF1 foci, in response to DNA DSBs

**Figure 4:**
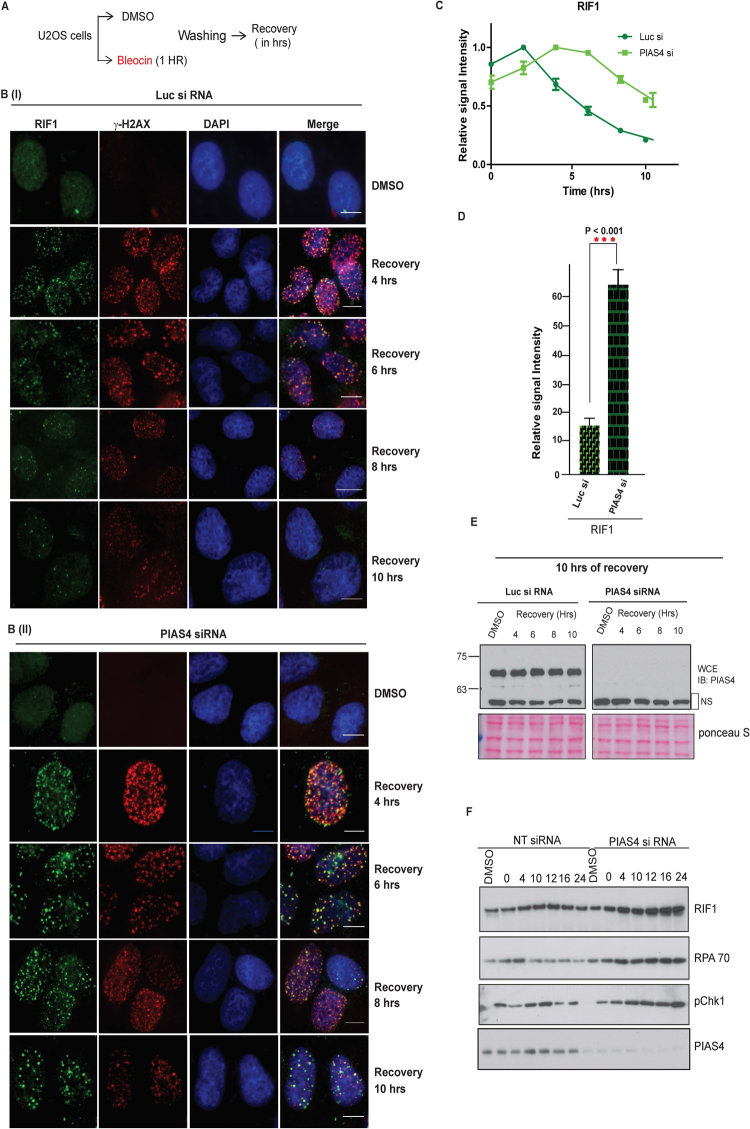
PIAS4 is required for the resolution of DNA damage induced RIF1 foci. (**A**) Schematic representation of experiment to assess the extent of resolution of DDR foci following recovery from DNA damage. (**B**) Representative images of U2OS cells were transfected with (i) control (Luc) siRNA and PIAS4 siRNA. Cells were treated with bleocin for 1 hour and recovered in fresh medium to allow for recovery from DNA damage. At the indicated timepoint following incubation in fresh media, cells were permeabilized and fixed with formaldehyde, followed by co-immunostaining with RIF1 and γ-H2AX antibodies. (**C**) Intensities of nuclear RIF1 signals were quantified using ImageJ and expressed as ratios of the maximal signal intensity detected within the 10 hours of recovery. Graph shows the intensity ratios of cells transfected with PIAS4-specific siRNA or control (Luc) siRNA at different timepoints following recovery. (**D**) As in (**B**) and (**C**), the relative signal intensities were detected using (i) RIF1 and γ-H2AX antibodies and quantified using ImageJ. Relative signal intensity ratios for the 10 hours timepoint were plotted, comparing control (Luc) siRNA and PIAS4 siRNA transfected cells. Data represent mean and SD from two independent experiments (*) P < 0.001. (**E**) WCE were immunoblotted to determine the knockdown level PIAS4 in experiment A to D. (**F**) Control (Luc) and PIAS4 siRNA transfected cells were treated with bleocin and harvested at the indicated timepoints following recovery in fresh media. WCE were immunoblotted using antibodies against RIF1, RPA70, pChk1 and PIAS4.

To investigate the importance of PIAS4-dependent RIF1 SUMOylation, we examined the recruitment of RIF1 to γH2AX-marked sites of damage in PIAS4-depleted cells. We observed a substantial increase in γH2AX foci that colocalized with Rif1 foci upon bleocin treatment (Fig. S4A). RIF1 foci also colocalize with pATM foci. (Fig. S4B). Despite an initial delay in the recruitment of RIF1 and γH2AX to DDR sites in PIAS4-depleted cells (Fig. 4C), we did not observe any drastic change in RIF1 or γH2AX foci formation (Fig. 4B-II and 4C), suggesting that the recruitment of RIF1 to sites of DNA damage is not completely dependent on PIAS4 functions. This prompted us to investigate if the dynamics of RIF1/DDR foci following recovery from DNA damage may be dependent on PIAS4. Therefore, we examined the resolution of RIF1 and γ-H2AX foci following recovery of cells from DNA damage (Fig. 4A and B). As expected, in control cells, the kinetics of RIF1/γH2AX foci shows a time-dependent resolution. Surprisingly, a striking contrast in the resolution of RIF1 foci was observed in PIAS4-depleted cells (Fig. 4BII and C). We observed a four-fold increase in the signal intensity of RIF1 foci remaining in PIAS4-depleted cells compared to control cells at 10 hours after recovery from bleocin (Fig. 4D), suggesting that PIAS4 depletion significantly impaired the clearance of RIF1 from DNA damage sites. PIAS4 depletion was tested by immunoblotting (Fig. 4E). Similarly, we noticed an increased RIF1, RPA70 and pCHK1 proteins, indicating an overall increase in DNA damage in PIAS4 depleted cells (Fig. 4F). Taken together, our data suggest that PIAS4 function is required for the disassembly of RIF1 DDR foci from the sites of DNA damage.

### PIAS4 prevents DNA double stand breaks in S phase cells and Ultrafine Bridges

**Figure 5:**
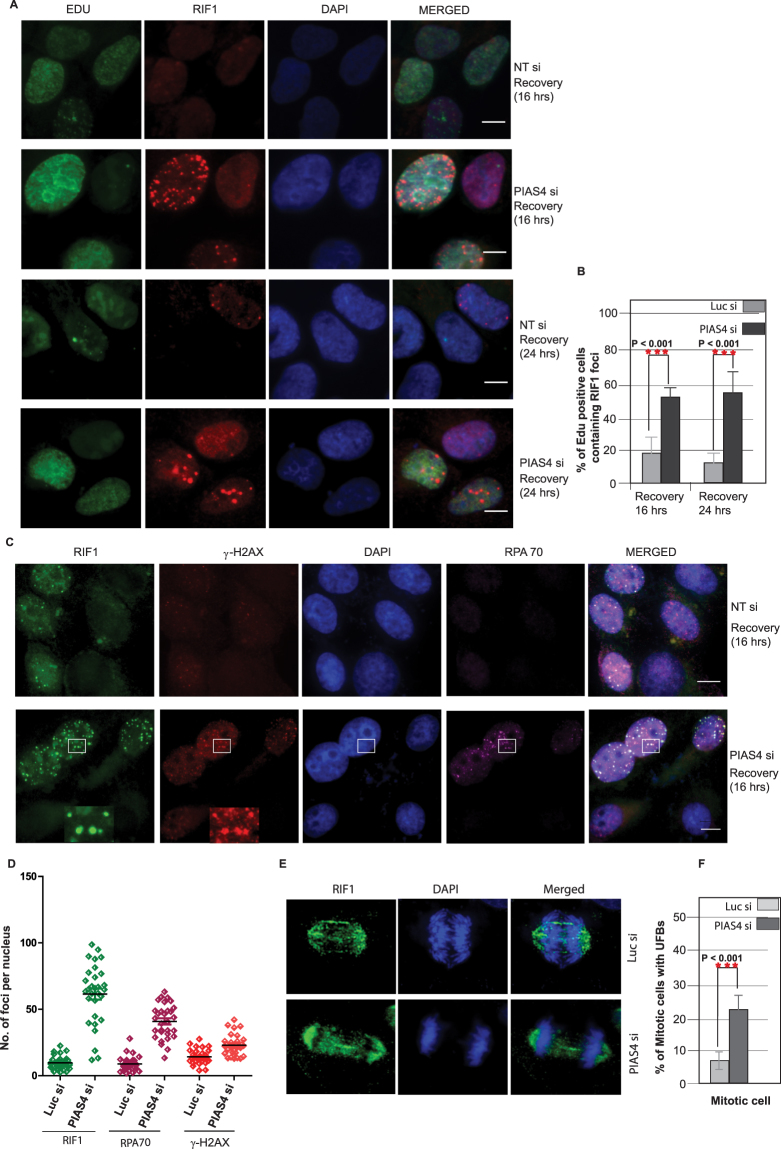
PIAS4 depletion resulted in persistent RIF1 foci and replication stress. (**A**) U2OS cells were transfected with control siRNA or PIAS4 siRNA and treated with bleocin (1 hr) or DMSO as control. Cells were recovered in fresh media for 16 hours or 24 hours. Formaldehyde fixed cells were immunostained with RIF1 antibody and EdU positive S phase cells were detected using Click-iT® assay kit. (**B**) Percentage of Edu positive cells displaying RIF1 foci at 16 hrs. or 24 hrs. following drug recovery, either in presence (Luc si) or in the absence of PIAS4 (PIAS4 si). (**C**) Control (Luc) siRNA and PIAS4 siRNA transfected U2OS cells were treated with bleocin (1 hr) and recovered in fresh medium for 16 hours. Cells were immunostained for RIF1, RPA70 and γ-H2AX. Inset shows colocalized RIF1, RPA70 and γ -H2AX foci. (**D**) After the recovery, number RIF1, γ-H2AX and RPA foci per cell were counted. (**E**) U2OS cells were transfected with control (Luc) siRNA or PIAS4 siRNA and immunostained with RIF1 antibody. Mitotic cells were analyzed for the presence of RIF1-positive ultrafine anaphase bridges (UFBs). (**F**) Percentage of mitotic cells with UFBs in control or PIAS4 siRNA transfected cells. Data represent at least two independent experiments; values represent mean ± SD.

The persistent nature of the RIF1 foci in PIAS4-depleted cells led us to further question the fate and consequence of these RIF1 foci. To investigate that, we extended the recovery time and examined the resolution of RIF1 foci at later time points. Consistently, most of the RIF1 foci were effectively resolved in cells having normal PIAS4 expression. However, we still observed intense RIF1 foci in PIAS4 depleted cells despite extended recovery (16 and 24 hours) (Fig. 5A). Interestingly, in the absence of PIAS4, we observed persistent RIF1 foci in S phase, as marked by EdU positivity (Fig. 5A). A careful quantification of EdU stained cells revealed that a substantial proportion of replicating cells harbor RIF1 foci in the absence of PIAS4 (Fig. 5B). Interestingly we noted distinct RPA70 foci formation in the absence of PIAS4 (Fig. 5C). A careful quantification revealed that RPA foci are clearly increased in PIAS4 depleted cells, compared to control cells, at the 16 hours of time point following recovery from bleocin. Secondly, a large proportion of these foci colocalized with the RIF1 foci and to some extent with γ-H2AX foci (Fig. 5D). Consistently, we observed a significant increase in RIF1 foci in PIAS4-depleted cells, compared with the control cells. Therefore, it is conceivable that one of the major consequences of PIAS4 absence is unresolvable RIF1 DDR foci, which persist longer and posing a potential threat to genomic integrity.

Recently, RIF1 has been reported to be recruited at Ultrafine Bridges (UFBs), which is a major consequence of abnormal DNA structure, carried-over from S-phase into mitosis^43^. During anaphase RIF1 promotes the resolution of UFBs and prevents these structures or chromosomal lesions from being transmitted to G1 cells in the form of nuclear bodies^44^. Consistent with this observation, we noticed RIF1 at the sites of UFBs in mitosis (Fig. 5E). We further investigated the involvement of PIAS4 in the regulation of the UFBs. Surprisingly, the frequency of UFBs formation was considerably increased in PIAS4 depleted cells (Fig. 5F). These observations strongly suggest that cells lacking PIAS4 activity are susceptible to replication stress, frequent UFBs formation and subsequently lead to genomic instability.

### PIAS4 prevents unusual RIF1 and 53BP1 nuclear bodies

**Figure 6:**
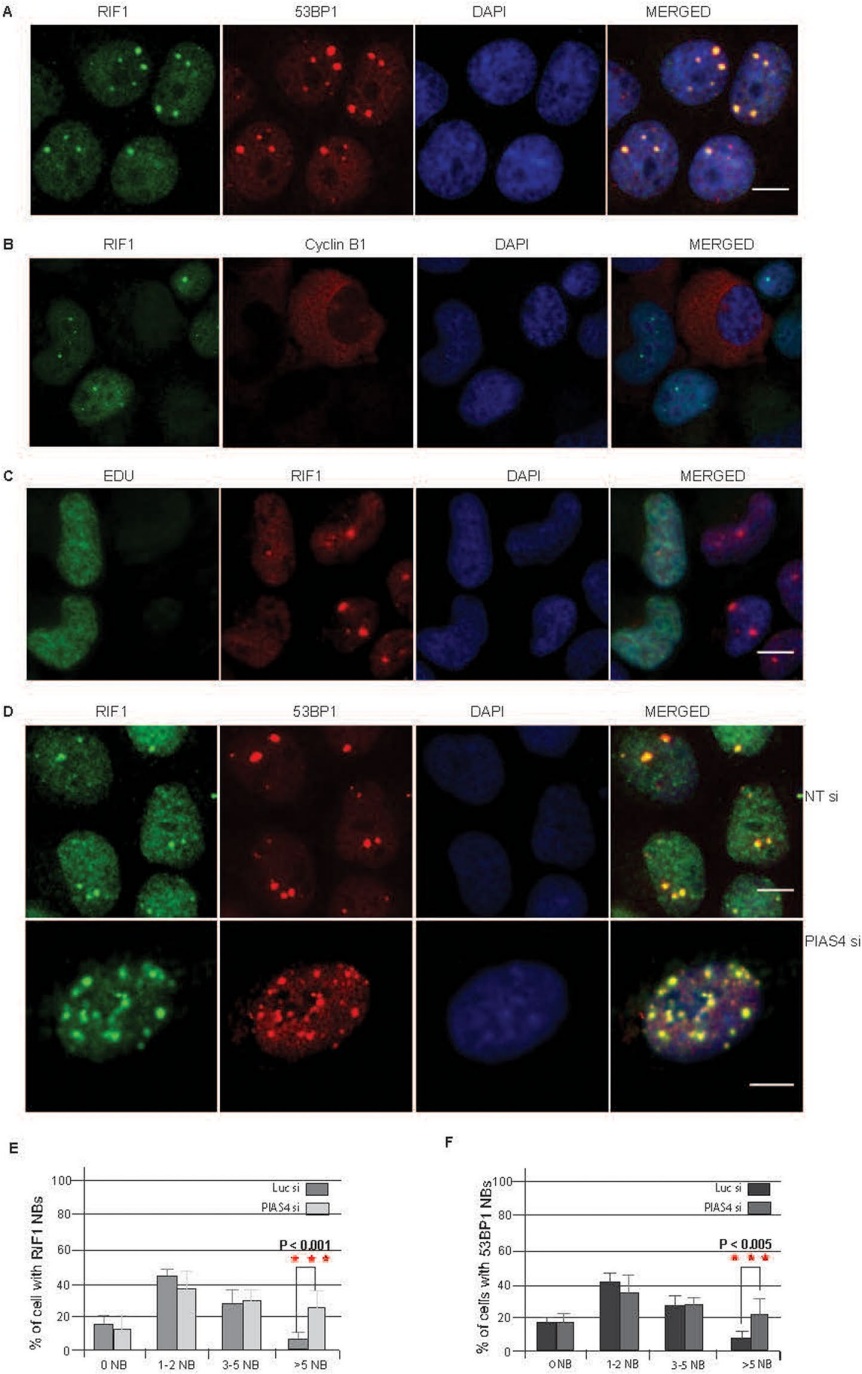
PIAS4 is required to suppress multiple RIF1/53BP1 Nuclear Bodies. (**A**) Nuclear bodies containing RIF1 and 53BP1 were detected using specific antibodies. (**B**) U2OS cells were co-immunostained with RIF1 and Cyclin B1 antibodies. (**C**) U2OS cells were incubated with EdU for 60 minutes and immunostained with RIF1 antibody. (**D**) U2OS cells transfected with either control siRNA or PIAS4 siRNA were immunostained with RIF1 and 53BP1 antibodies. (**E**) Percentage of EdU positive cells containing different numbers of RIF1 nuclear bodies either in presence of in the absence of PIAS4. (**F**) Percentage of Edu positive cells containing different numbers of 53BP1 nuclear bodies either in presence of in the absence of PIAS4. Data represent at least two independent experiments; values represent mean ± SD.

Unresolved replication intermediates generated during late stage of replication, can lead to the formation of nuclear bodies in G1 cells^44–46^. In normal proliferating cells, the p53 binding protein 53BP1 has been identified as a key component of these nuclear bodies^45–47^. Upon induction of low dose of replication stress, 53BP1 differentially colocalize to MDC1, pATM, RNF168, Ubiquitin conjugating enzyme (FK1), BLM and few other proteins involved in DNA replication stress. We consistently noticed intense and enlarged RIF1 foci resembling the previously described 53BP1 nuclear bodies (Fig. 6A). The previously reported accumulation of 53BP1 and other DNA Repair proteins in nuclear bodies (NBs), prompted us to examine the colocalization of RIF1 with NBs. Indeed, a majority of RIF1 colocalize with 53BP1-positive NBs (Fig. 6A). To determine the cell cycle specificity, cells were immunostained with RIF1 and CyclinB1 antibodies (listed in Table 3). We noticed that RIF1 NBs were mainly present in Cyclin B1 negative cells, indicating that these specific foci are absent in G2 cells (Fig. 6B). We further confirmed our observation using EdU labeling of S phase cells and noticed that RIF1 NBs were present predominantly in EdU negative cells (Fig. 6C). Together, these data suggest that, similar to previously identified 53BP1, RIF1 also exist as nuclear bodies in G1 cells.

**Table 3:** List of Antibodies.

Antibody	Species	Supplier	Cat. No.
Anti-beta-Actin	mouse	Thermo scientific	MA5-15739
Anti-RIF1	rabbit	Bethyl	A300-569A
Anti-RIF1	rabbit	Bethyl	A300-568A
V5 Tag	Mouse	Thermo Scientific	R960-25
Anti-BLM	Goat	SantaCritz	Sc-7790
Anti-γ-H2AX (S139)	mouse	Millipore	05-636
Anti-PIAS1	rabbit	Cell Signalling Technology	D33A7
Anti-PIAS4	rabbit	Cell Signalling Technology	D2F12
Anti-RNF4	rabbit	Eurogentec	Custom made
Anti-SUMO2/3	mouse	Abcam	ab81371
Anti-SUMO2/3	Rabbit	Eurogentec	Peptide 2277
Anti-UBC9	mouse	BD Transduction Labs	610748
Anti-53BP1	mouse	MAB3802	Millipore
Anti-53BP1	mouse	Sc 22760	SantaCrutz
Cyclin B1	mouse	BD pharminogen	554177
RPA 70	Rat	Cell Singalling	#2267
Anti-Goat Alexa fluor 488	Donkey	Life-Tech	A-11055
Anti-mouse Alexafluor 488	Donkey	Life-Tech	A-21202
Anti-rabbit Alexa fluor 488	Donkey	Life-Tech	A-21206
Anti-rabbit Alexa fluor 594	Donkey	Life-Tech	A-21207
Anti-mouse Alexa fluor 594	Donkey	Life-Tech	A-21203
Anti-rat Alexaflur 647	Goat	Life-Tech	A-21244.

Given that nuclear bodies are a potential consequence of aberrant UFBs resolution^45^ and our data suggests that PIAS4 plays a critical role in suppressing abnormal UFBs formation (Fig. 5E and F), we predicted that PIAS4 might have important role in preventing the formation of NBs. Indeed, we found that PIAS4 depleted cells displayed a remarkable increase in the number of intense NBs, containing both RIF1 and 53BP1 (Fig. 6D). Considering the differential number of nuclear bodies in each cell, we grouped cells according to the number of NBs present in different cells (Fig. 6E). Interestingly, compared to control siRNA treated cells, the proportion of cells with more than 5 nuclear bodies were increased in PIAS4 depleted cells (Fig. 6E and F). Together, these data suggest an important role of PIAS4 in the suppression of RIF1 and 53BP1 nuclear bodies.

### PIAS4 is required for RIF1 Ubiquitination

**Figure 7:**
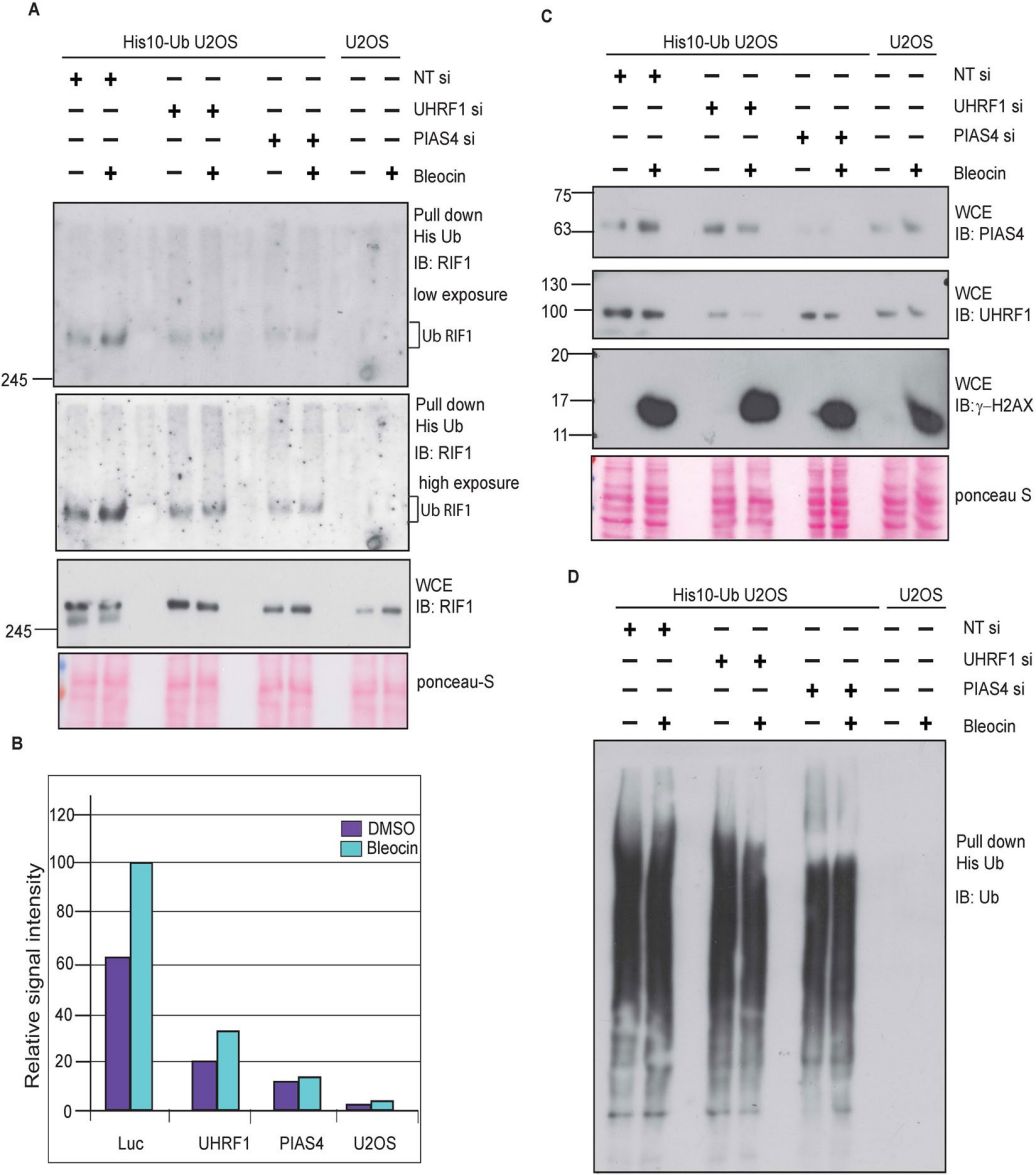
PIAS4 regulates RIF1 Ubiquitination. (**A**) U2OS cells stably expressing His10-Ubiquitin were transfected with control (Luc) siRNA or siRNAs specific to UHRF1 or PIAS4. Cells were treated with bleocin or DMSO as control and harvested for His purification. His purified Ubiquitin conjugates were immunoblotted with RIF1 antibody, and anti-His and anti-Ub to detect total His/Ub pulldown in all conditions. (**B**) The intense ubiquitinated RIF1 protein bands were quantified by imageJ software and relative image signal intensity of different protein samples were plotted on Y-axis. (**C**) Protein samples from WCE were immunoblotted with indicated antibodies to detect the expression of RIF1 (Fig. 7A lower panel), PIAS4, UHRF1 and y-H2AX (Fig. 7C). (**D**) His ubiquitin conjugates were immunoblotted with ubiquitin specific antibody to determine the level of total ubiquitin in different protein samples.

Epigenetic regulator UHRF1 (Ubiquitin-like, with PHD and RING finger domains 1) has been shown to play important role in the maintenance of genomic integrity. Recently, it has been shown that UHRF1 is recruited by BRCA1 in S phase to ubiquitinate RIF1 and promotes its dissociation from 53BP1 at DNA DSB sites^48^. Here, we show that RIF1 foci persist in EdU positive cells in the absence of SUMO E3 ligase PIAS4. These results prompted us to question if RIF1 ubiquitination and protein turnover at sites of DNA breaks has been compromised, specifically when PIAS4-dependent SUMOylation of RIF1 is impaired. We hypothesized that PIAS4 mediated RIF1 SUMOylation is required for RIF1 ubiquitination and dissociation from the site of DNA damage. To analyze the RIF1 Ubiquitination *in vivo*, U2OS cell stably expressing 10-His Ubiquitin were treated with bleocin. Consistently, we detected RIF1 ubiquitination that was largely dependent on UHRF1. Remarkably, we also noticed a substantial reduction in the extent of RIF1 Ubiquitination when PIAS4 is depleted (Fig. 7A). The level of ubiquitinated RIF1 signal intensity in UHRF1 depleted cells was comparable to that in PIAS4-depleted cells (Fig. 7B). Whole cell lysates were analyzed to detect the protein levels of PIAS4, UHRF1 and γ-H2AX in damaged and untreated cells (Fig. 7C). His purified Ubiquitin protein conjugates were immunoblotted to determine the total level of ubiquitin (Fig. 7D).

### C-Terminal region of RIF1 is SUMOylated

**Figure 8:**
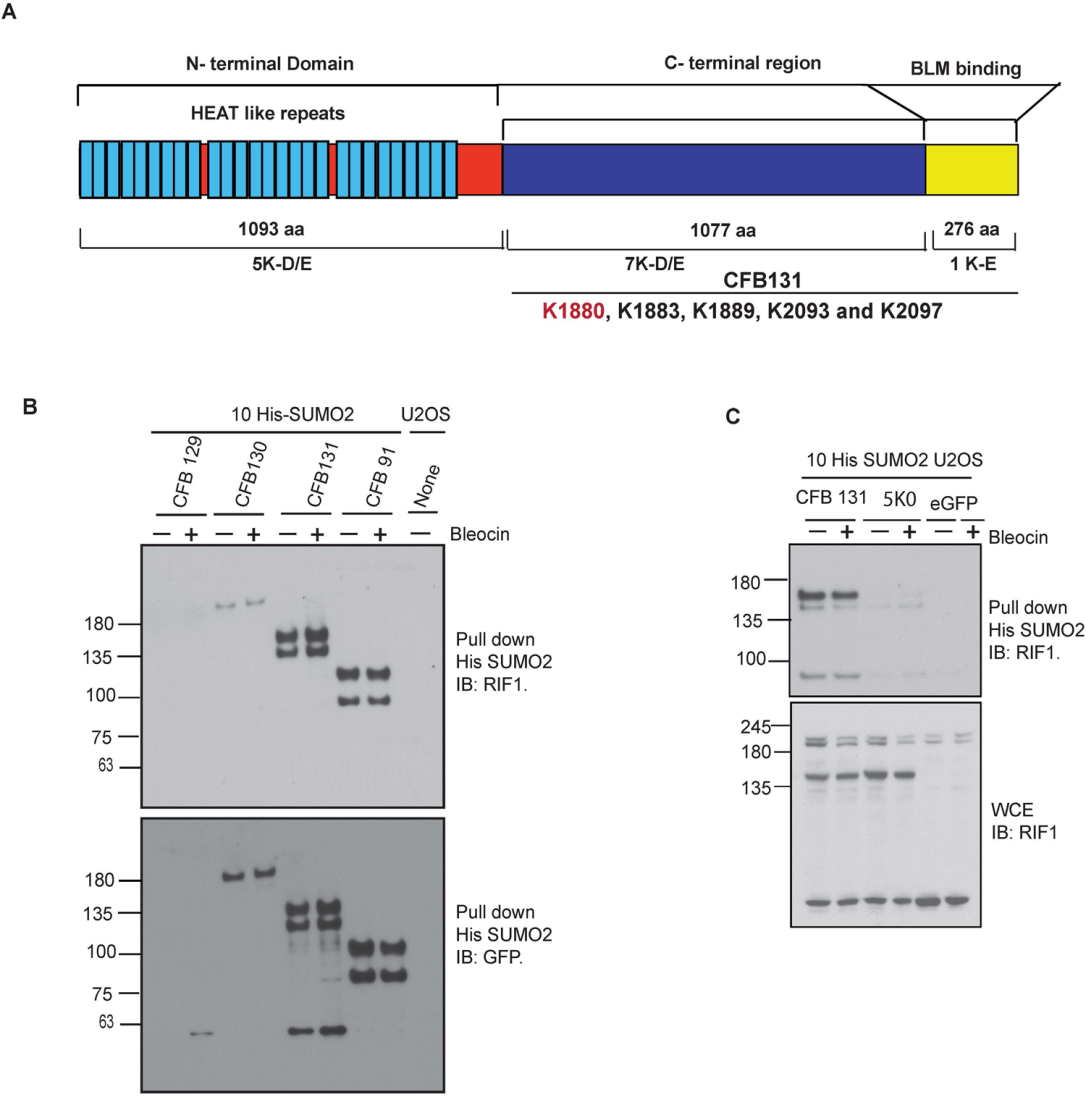
RIF1 C-terminal region is efficient for SUMOylation. (**A**) Schematic presentation of Linear structure of RIF1 protein. (**B**) U2OS cells stably co-expressing His10-SUMO2 and indicated GFP fused RIF1 fragments were either DMSO treated or treated with Bleocin. Cells were harvested and His purification was performed. His purified SUMO2 conjugates were resolved in 3–8% Tris-acetate gel and immunoblotted with anti-RIF1 (upper panel) and ant-GFP (lower panel) and SUMO2/3 antibody (lowest pane). (**C**) U2OS cells stably expressing  His10-SUMO2 were transiently transfected with GFP fused RIF1 fragment (CFB131), Lysine deficient mutant RIF1 fragment (5K0) and an empty vector. After indicated treatment cells were harvested and His purification was performed with the Lysed samples. WCE and His purified SUMO2 conjugates were separated in 4–12% Bis-Tris gel and immunoblotted with RIF1 antibody.

**Figure 9:**
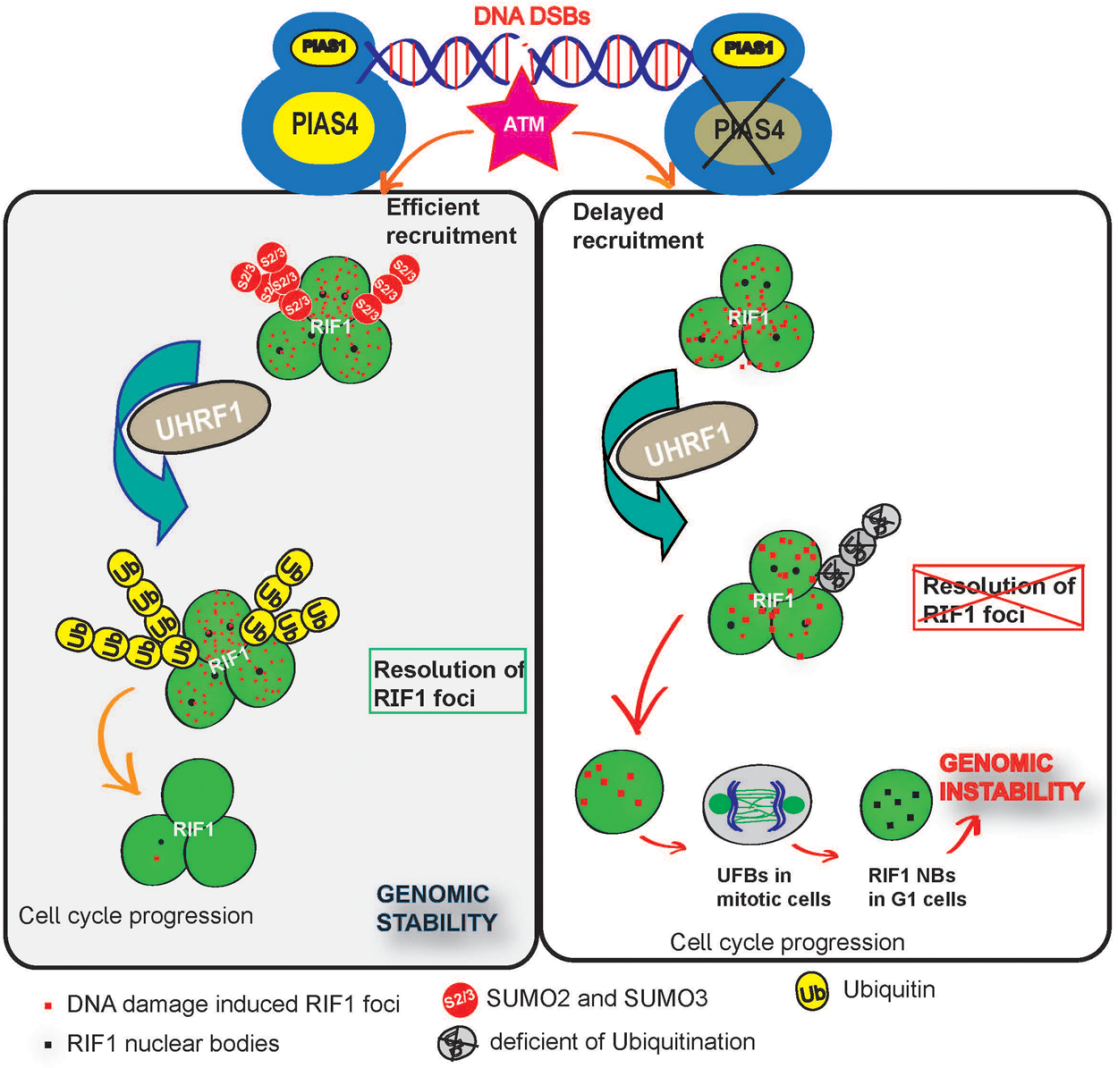
MODEL: SUMO E3 Ligase PIAS4 maintains genomic stability. SUMO E3 ligases PIAS4 and PIAS1 are central to DDR signaling. In normal conditions, DNA damage induced 53BP1 and RIF1 foci colocalize with y-H2AX. SUMOylated 53BP1 and RIF1 facilitate efficient recruitment, break repair and removal of RIF1 from the sites of DNA breaks. Timely resolution of RIF1 coupled to the cessation of DDR signals and y-H2AX foci allows cells to resume normal cell cycle progression, thus maintaining overall genomic stability. In the absence of PIAS4, RIF1 SUMOylation is impaired, resulting in decreased RIF1 ubiquitination and it's  dissociation from  the sites of DNA dmage. Unresolved RIF1 foci accumulate even as cells enter S phase. Consequently, enhanced UFBs are also detected in mitotic cells depleted of PIAS4 that potentially leads to the formation of multiple RIF1 nuclear bodies in the subsequent G1 cell cycle phase. Taken together, our findings support a model in which PIAS4 is needed to promote genomic integrity by promoting the timely clearance of RIF1 from sites of repaired DNA breaks.

Biochemical analysis of BLM protein complex revealed Rif1 as an important component. BLM binding of Rif1 is absolutely dependent on its conserved C-terminal domain and is independent of the N-terminal Heat repeat region of Rif1^37^. A domain structure of RIF1 is shown in Fig. 8A. To determine the potential sites of RIF1 SUMOylation, we obtained different deletion fragments of RIF1^25^ and established stable cell lines of GFP-fused RIF1 fragments expressing, aa 406–2446 (CFB129), aa 1355–2446 (CFB130), aa 1701–2446 (CFB 131) and aa 1924–2446 (CFB 91) in 10-His-SUMO2 U2OS background. His purified SUMO2 conjugates were immunoblotted either with antibodies raised against RIF1 or GFP. We demonstrated that CFB131 was the most efficiently SUMOylated fragment compared to other deletion fragments (Fig. 8B). Bioinformatic tool and online available database suggest that a majority of consensus SUMOylation sites are clustered in C-terminal region of the protein. Four potential SUMO acceptor Lysine sites (K1883, K1889, K2093 and K2097) are found in CFB131. In addition, a previous study using mass spectrometry analysis shown that another Lysine residue, K1880, is SUMOylated^49^. Therefore, we mutated all five potential SUMO acceptor lysine residues in the CFB131 fragment. Interestingly, we found that RIF1 SUMOylation was significantly reduced in Lysine deficient (5K0) mutant (Figs 8C & S8), strongly suggesting that one or more of these five lysine sites could be SUMOylated. Overall, our results indicate that PIAS4 mediated RIF1 SUMOylation play important role in the maintenance of Genomic stability (Fig. 9).

## Discussion

Coordinated protein SUMOylation and ubiquitination are key PTMs required for the recruitment and repair of DSBs in a timely manner^11,50,51^. SUMO E3 Ligases PIAS1 and PIAS4 have been shown to play important roles in the SUMO modification of target proteins^12,15^. We identified PIAS4 as a critical regulator of RIF1 SUMOylation. Earlier studies have shown that the DNA damage response mediator proteins BRCA1, 53BP1 and MDC1 are SUMOylated in response to genotoxic stress^12,18,20^. In this study we have identified RIF1 as a SUMOylated protein. RIF1 SUMOylation is increased in G1 cells and enriched in response to different DNA damaging agents, suggesting a broader role of RIF1 SUMOylation to counteract genotoxic stress. In addition to RIF1, we also demonstrated that 53BP1 protein is SUMO2 modified. Cell cycle synchronization study further highlights that both RIF1 and 53BP1 are strongly SUMOylated in G1 and extent of SUMOylation was increased in response to bleocin treatment. Our data suggesting that DNA damage induced RIF1 foci strongly colocalize with SUMO2 and to some extent with SUMO3, but not with SUMO1. Together our data strongly suggest that RIF1 is predominantly modified by SUMO2/3.

Earlier findings suggest that PIAS4 regulates both SUMO1 and SUMO2/3 accrual in laser tracks^12^. Here we identified an additional DDR protein RIF1, as a PIAS4 target for SUMOylation. Consistent with earlier findings^12^, we noticed only a partial loss of 53BP1 SUMOylation after PIAS1 knockdown and a substantial loss was observed after PIAS4 knockdown, suggesting that PIAS4 is the main SUMO E3 ligase required for the 53BP1 SUMOylation. Although it is a possibility that PIAS4 may regulate the function of RIF1 via 53BP1, given that many reports have demonstrated that RIF1’s function in NHEJ is regulated by 53BP1^26–32,52^, our data clearly show RIF1 SUMOylation is not affected by 53BP1 depletion. This strongly suggests that PIAS4 promote the SUMOylation of 53BP1 and RIF1 independently, and argues for a direct role of PIAS4-dependent SUMOylation of RIF1. Coimmunoprecipitation experiment suggest a stronger binding of RIF1-PIAS4 in G1 cells compared to G2 cells, and supports our finding that RIF1 is mainly SUMOylated by PIAS4 in G1. In addition, we also noticed a DNA damage responsive interaction of RIF1 and PIAS4 in S phase cells, indicating other potential role of PIAS4 in regulating RIF1 in replicating cells, a subject of future investigation.

In addition to known consequences of PIAS4 depletion, our results suggest new roles in the regulation of DNA damage induced RIF1 foci resolution. RIF1 is recruited at the sites of DNA damage and colocalize with different DDR signaling proteins including γ-H2AX. In accordance with the role of PIAS4 in DDR signaling, we observed a clear delay in the accumulation of DNA damage induced RIF1 and γ-H2AX foci in PIAS4-depleted cells. Importantly, we found that RIF1 foci was persistent even at 16–24 hours of DNA damage recovery when PIAS4 was depleted. Therefore, we speculate that the persistence of RIF1 at sites of DNA damage in the absence of PIAS4, indicate that DNA damage induced PIAS4 dependent RIF1 SUMOylation is essentially required for the complete removal of RIF1 from the sites of DNA breaks post repair.

The peculiar nature of the unresolved RIF1 foci prompted us to analyze these cells at longer time points, followed by recovery from acute DNA damage. We observed a large number of S phase cells harboring RIF1 foci. The distinct RPA70 foci colocalization with RIF1 strongly suggesting that SUMO E3 ligase activity of the PIAS4 is required for the removal of RIF1 from the site of DNA breaks. This raises important questions as to how cells deal with unresolved RIF1 during replication and if these unresolved foci may pose impediments to DNA replication which can risk the genomic stability. In line with this, we demonstrated that PIAS4 activity is needed to prevent the occurrence of UFBs, which are known to result from replication problems and are associated with the loci that are either under-replicated and/or associated with abnormal DNA structures. Lack of PIAS4 increases the incidence of UFBs (>3 fold). We speculate that lack of RIF1 removal from chromatin may impede normal replication and result in abnormal replication of genomic regions that are visualized as UFBs in mitosis. In PIAS4 depleted cells, we also detected an increase in RIF1/53BP1 nuclear bodies. We described here for the first time that RIF1 is present in 53BP1-positive nuclear bodies, which are thought to be chromatin lesions that are transmitted from mitosis to G1^45,46^. Accordingly, aberrant UFB dissolution in mitosis may cause the accumulation of nuclear bodies in the subsequent G1 phase. Our data suggest that the increase in nuclear bodies that comprise of RIF1 and 53BP1 in PIAS4 depleted cells may result from the increase in UFBs and, most likely replication problems. Interestingly, recently it has been shown that RIF1 plays a 53BP1 independent role in the maintenance of genomic stability through its recruitment at UFBs and its resolution^44^. Currently we cannot exclude the possibility that PIAS4 mediated RIF1 function may be directly required for the resolution of UFBs. Therefore, we propose that PIAS4 activity is required to suppress the formation of 53BP1 and RIF1 nuclear bodies.

Recently UHRF1 has been identified as downstream target of BRCA1. In S phase cells UHRF1 ubiquitinates RIF1 and promotes its dissociation from 53BP1, which in turn promotes HR^48^. Our study suggests that PIAS4 mediated SUMO2/3 modification of RIF1 is a primary event, required for efficient break repair and removal of RIF1 from the sites of DNA damage by promoting the ubiquitination of RIF1. Further studies are required to elucidate the mechanism of how SUMOylation of RIF1 may directly influence the ubiquitination of RIF1. In addition, further studies are required to investigate the role of PIAS4 in the regulation of UHRF1 activity.

Our data suggest that one or more of the five lysine residues located in the C-terminal region of the protein, required for RIF1 SUMOylation. Indeed, one of the site (K1880) has already been reported to be SUMOylated *in vivo*^49^. Lysine1880 is located in the motif ETKEEKPEETP, contains plenty of glutamic acids in the region and two prolines, indicating an unstructured region in the protein most likely located at the surface as a result of the charged side chains. Overall, our study proposes a model in which PIAS4 dependent regulation of RIF1 SUMOylation is required for efficient DDR signaling and resolution of RIF1 from sites of DNA damage in a timely manner. PIAS4 deficiency therefore results in genomic instability, as evidenced by the rise of DNA DSBs, accumulation of UFBs in mitotic cells and RIF1 nuclear bodies in G1 cells (Fig. 9).

## Method

### Plasmid DNA

YFP-SUMO1, RFP-SUMO2 and RFP-SUMO3 plasmid DNA constructs are a kind gift from Prof. Ron Hay Lab. FLAG-PIAS4 (add gene #15208).

### Lentiviral transduction

The source of shRNA clones are listed in Table 4. One million cells were seeded in a 15-cm dish and the next day, cells were either infected with shRNA viruses directed against PIAS1 and PIAS4 or control non-targeting shRNA viruses at MOI 2. After changing media on the third day, cells were incubated for another 3 to 4 days before the experiment.

**Table 4:** List of shRNA constructs.

Target gene	TRC No. or ID no.
RIF1 (I)	TRCN0000154312
RIF1 (II)	TRCN0000155531
PIAS1 (I)	TRCN0000004145
PIAS1 (II)	TRCN0000004147
PIAS4 (I)	TRCN0000004115
PIAS4 (II)	TRCN0000004118
None (Scrambled shRNA)	Add gene Plasmid #1864

### siRNA transfection

The siRNA duplexes were purchased from Dharmacon (listed in Table 2). 1.8 million cells were seeded in a 15 cm dish and reverse transfection was performed according to manufacturer’s instructions. 18 hours after the transfection, fresh growth medium was added. 72 hrs after the transfection, the indicated drug treatments were performed and cells were harvested.

### Cell culture and cell cycle analysis

U2OS cells and U2OS cells stably expressing His SUMO2 were grown in DMEM high glucose medium added with 10% FBS and 100 U/ml penicillin plus 100 µg/ml streptomycin (Life Technologies) at 37 °C with 5% CO2. To arrest cells at the G1/S cell cycle stage, cells were treated with 2 mM thymidine for 19 hrs and then released for 9 hours, followed by a second thymidine block for 18 hrs. To release G1-arrested cells, they were washed with PBS and prewarmed cell culture medium. Cells were collected after 4 hours and 8 hours of G1 release to obtain S-phase or S/G2 enriched population. After washing with PBS, cells were transferred into 70% ethanol and incubated for 30 minutes. Subsequently cells were incubated with Ribonuclease A and stained with propidium iodide^18^ for 20 minutes and analyzed by flow cytometry.

### Microscopy

Cells for immunofluorescence microscopy were cultured on square glass slides (22 × 22 mm) in 6 well plates. After treatment with MG132 (10 µM) for 5 hrs. and/or Bleocin (5 µg/ml) for 4 hours, U20S cell were washed 3 times in PBST (phosphate buffer saline containing 0.1% tween-20 detergent). Cells were incubated with ice- cold 2% formaldehyde (w/v) for 20 minutes and washed 3 times with PBST. Fixed cells were blocked with 5% BSA in PBST for overnight at 4-degree C. After blocking, cells were stained with appropriate antibodies (listed in Table 3) in PBST containing 5% BSA. Cells were washed 3 times in PBST and then co-immunostained with anti-rabbit or anti-mouse Alexa Fluor® 488 and anti-rabbit or anti-mouse Alexa Fluor® 594 for 30 minutes. Cell were washed 3 times with PBST and counter stained with DAPI. After mounting, imaging was performed with Zeiss AxioImager Z1 upright (EBL) Olympus microscope.

### Purification of SUMO-2 and Ubiquitin conjugates

His-SUMO-2 and Ubiquitin conjugates were purified essentially, as described previously (Vyas *et al*., 2013). U2-OS cells and stably expressing 10 His SUMO2 or 10 His-Ub, were washed and collected in ice-cold PBS. Small aliquots of cells were lysed in 1% SDS, 1% NP-40, 100 mM Tris/Hcl (pH 7.5), 150 mM Nacl to determine the protein concentration. Guanidinium lysis buffer (6 M guanidinium-HCl, 0.1 M Na2HPO4/NaH2PO4, 0.01 M Tris/HCl, pH 8.0, and competing imidazole) was added to the cell pellet to lyse the cells, after which, the cells were sonicated to reduce the viscosity. His-SUMO-2 conjugates were enriched on nickel-nitrilotriacetic acid-agarose beads (Qiagen) after which the beads were washed using wash buffers A to D. Wash buffer A: 6 M guanidinium-HCl, 0.1 M Na2HPO4/NaH2PO4, 0.01 M Tris/HCl (pH 8.0), 10 mM β-mercaptoethanol, 0.3% Triton X-100. Wash buffer B: 8 M urea, 0.1 M Na2HPO4/NaH2PO4, 0.01 M Tris/HCl (pH 8.0), 10 mM b-mercaptoethanol, 0.3% Triton X-100. Wash buffer C: 8 M urea, 0.1 M Na2HPO4/NaH2PO4, 0.01 M Tris/HCl (pH 6.3), 10 mM β-mercaptoethanol, 0.3% Triton X-100. Wash buffer D: 8 M urea, 0.1 M Na2HPO4/NaH2PO4, 0.01 M Tris/HCl, pH 6.3, 10 mM β-mercaptoethanol, 0.1% Triton X-100. Samples were eluted in 7 M urea, 0.1 M NaH2PO4/Na2HPO4, 0.01 M Tris/HCl (pH 7.0), 500 mM imidazole.

### RFP-TRAP Assay

Asynchronously growing U2OS cells were washed with PBS, trypsinized and resuspended in ice-cold PBS. 10 million cells were transferred into Protein LoBind Eppendorf tubes and spun down for 5′ at 1500 rpm. Cells were lysed in 0.3 ml EBC buffer (50 mM Tris pH 7.3, 150 mM NaCl, 0.5% NP-40, 1 mM MgCl2. 50 mM Idoacetamide) and sonicated. The Lysed protein samples were added with 500 U Benzonase (in 700 µl EBC buffer). Protein lysates were incubated for 60 min under rotation. NaCl concentration was made to 300 mM, by adding 30 µl 5 M NaCl to 1 ml lysate. spin down lysate for 10′ full speed in Eppendorf centrifuge. Equilibriate RFP-Beads (Chromotek RTA-20) in wash buffer. Transfer lysate to Eppendorf tube with 50 µl RFP-Trap A beads suspension. Incubate 1.5 hr under rotation. Wash 3x with EBC buffer without Iodoacetamides. Elute with 4X LDS buffer.

### Immunoblotting

Whole cell extracts (WCE) were lysed in RIPA buffer supplemented with competing amount of protease inhibitor. WCE or purified protein samples were first separated on Novex 4–12% gradient gels (Life Technologies) using MOPS buffer or on Novex 3–8% gradient gels (Life Technologies) using Tris-Acetate buffer and then transferred onto 0.45 NC Nitrocellulose blotting membrane (GE Healthcare; 10600003) using a submarine system (Life Technologies). Membranes were stained with Ponceau S to visualize total protein and blocked with PBST containing 5% milk powder and 0.01% Tween-20 before incubating with primary antibodies as indicated.

### Recovery Assay

U2OS cells were seeded at 50 k cell density on cover slips. Next day cells were either treated with Luc si RNA or PIAS4 si RNA. After 72 hours of siRNA transfection, cells were either mock treated or treated with 5 µM of bleocin. After one hour of bleocin treatment cells were washed 2 times with PBS, one time with pre-warmed media and started to recover in new media. Cells on coverslips were permeabilized and fixed at different time points. U2OS cells were grown in 6 well plates transfected either with Luc siRNA or PIAS4 siRNA. As described above, after 72 hours of transfection, recovery experiment was performed. Cells were lysed in RIPA buffer supplemented with competing concentration of protease inhibitor and immunoblotted with indicated antibodies.

### Edu staining

To determine the no. of S-phase cells, U2OS cells were added with 10 µM of the Edu dye for 30 minutes. Premetallized and formaldehyde fixed cells were stained with RIF1 primary antibody and respective secondary antibodies according to Invitrogen- Click IT Edu Alexa Fluor 488 kit’s instructions.

### Cell Imaging and data analysis

All microscopic imaging was done in at 63X oil immersion objective Lens in Olympus Zeiss AxioImager Z1 upright fluorescence microscope. The image processing and quantification was primarily done with the help of FIJI and ImageJ software.

## Electronic supplementary material

Supporting Information
