# Author Correction: Dynamics of RIF1 SUMOylation is regulated by PIAS4 in the maintenance of Genomic Stability

**DOI:** 10.1038/s41598-018-23385-4

**Published:** 2018-03-22

**Authors:** Ramesh Kumar, Chit Fang Cheok

**Affiliations:** 1 IFOM-p53Lab Joint Research Laboratory, 8A Biomedical Grove, #06-38, Immunos, A*STAR, S138648 Singapore, Singapore; 2 IFOM, The FIRC Institute of Molecular Oncology Foundation, Via Adamello 16, 20139 Milan, Italy; 3 Department of Biochemistry, Yong Loo Lin School of Medicine, National University of Singapore, Singapore, S117597 Singapore; 4 Department of Pathology, Yong Loo Lin School of Medicine, National University of Singapore, Singapore, S119077 Singapore; 5 Cancer and Stem Cell Biology Program, Duke–NUS Graduate Medical School, 8 College Road, Singapore, 169857 Singapore

Correction to: *Scientific Reports* 10.1038/s41598-017-16934-w, published online 12 December 2017

The original version of this Article contained a typographical error in the spelling of the author Chit Fang Cheok, which was incorrectly given as Cheok Chit Fang. This has now been corrected in the PDF and HTML versions of the Article, and in the accompanying Supplementary Material.

