# Supplementary material for: Dynamics of RIF1 SUMOylation is regulated by PIAS4 in the maintenance of Genomic Stability
Source: Sci Rep. 2017 Dec 12;7:17367. doi: 10.1038/s41598-017-16934-w (PMC5727183; doi:10.1038/s41598-017-16934-w)
Supplement: Supplementary file 1 — Supporting Information [file 41598_2017_16934_MOESM1_ESM.docx]

**Supplementary Files:**

**Dynamics of RIF1 SUMOylation is regulated by PIAS4 in the maintenance of Genomic Stability.**

Ramesh Kumar^1-2^ Chit Fang Cheok^1-5^

**Figure legends:**

**Figure S1: Related to main figure 1**

Related to Figure1: (A) WCE and His purified protein samples from main figure 1A were immunoblotted with RIF1 and SUMO2/3 antibody. (B) WCE and His purified protein samples from unsynchronized and G1 synchronized cells were separated in 4-12% Bis-Tris Gel and immunoblotted with 53BP1 and SUMO2/3 antibody. (C) U2OS cell were transiently transfected with YFP fused SUMO1 and indicated treatment was performed after 48 hours. Cell were incubated with RIF1 primary antibody and an Alexa Fluor 594 secondary antibody.

**Figure S2: Related to main figure 3**

Related to Figure3: (A) Experiment described in figure 3A was repeated independently with siRNAs directed to deplete either PIAS1 and PIAS4. His purified proteins were immunostained with anti-His (B) and anti-SUMO2/3 antibodies (C).

**Figure S3: Related to main figure 4.**

Related to main figure 5: U2OS cells grown on glass coverslips, were primarily treated with control siRNA or PIAS4 siRNA. After 3 days, cells were either DMSO treated or treated with bleocin for 4 hours. After permeabilization step, formaldehyde fixed cells were co-immunostained with RIF1 and γ-H2AX antibody (A), RIF1 and pATM (B). U2OS cells were stained with RIF1 and p53BP1 antibody and after washing counterstained with Alexa Fluor 488 and 594 respectively and, nuclear staining with DAPI. Cells were subsequently incubated in mounting media and analyzed by microscopy (C).

**Figure S4:** **Related to main figure 8**

Related to main figure 8: As described in figure 8, GFP-fused RIF1 fragments expressing wild type C-terminal region of the RIF1 (CFB131) and two independent clones of Lysine deficient RIF1 fragment (5K0), were transiently transfected in10-His SUMO2 expressing U2OS cell line. His purified SUMO2 protein conjugates were immunoblotted with anti-RIF1 and anti-GFP antibodies (upper panel). Whole cell lysates were immunoblotted with SUMO2/3 antibody (lower panel)
